# Water–fat separation in spiral magnetic resonance fingerprinting for high temporal resolution tissue relaxation time quantification in muscle

**DOI:** 10.1002/mrm.28143

**Published:** 2020-01-03

**Authors:** Kirsten Koolstra, Andrew G. Webb, Thom T. J. Veeger, Hermien E. Kan, Peter Koken, Peter Börnert

**Affiliations:** ^1^ C.J. Gorter Center for High Field MRI, Radiology Leiden University Medical Center Leiden Netherlands; ^2^ Philips Research Hamburg Germany

**Keywords:** conjugate phase reconstruction, exercise, fat, magnetic resonance fingerprinting, muscle, spiral

## Abstract

**Purpose:**

To minimize the known biases introduced by fat in rapid T_1_ and T_2_ quantification in muscle using a single‐run magnetic resonance fingerprinting (MRF) water–fat separation sequence.

**Methods:**

The single‐run MRF acquisition uses an alternating in‐phase/out‐of‐phase TE pattern to achieve water–fat separation based on a 2‐point DIXON method. Conjugate phase reconstruction and fat deblurring were applied to correct for *B*_0_ inhomogeneities and chemical shift blurring. Water and fat signals were matched to the on‐resonance MRF dictionary. The method was first tested in a multicompartment phantom. To test whether the approach is capable of measuring small in vivo dynamic changes in relaxation times, experiments were run in 9 healthy volunteers; parameter values were compared with and without water–fat separation during muscle recovery after plantar flexion exercise.

**Results:**

Phantom results show the robustness of the water–fat resolving MRF approach to undersampling. Parameter maps in volunteers show a significant (*P* < .01) increase in T_1_ (105 ± 94 ms) and decrease in T_2_ (14 ± 6 ms) when using water–fat‐separated MRF, suggesting improved parameter quantification by reducing the well‐known biases introduced by fat. Exercise results showed smooth T_1_ and T_2_ recovery curves.

**Conclusion:**

Water–fat separation using conjugate phase reconstruction is possible within a single‐run MRF scan. This technique can be used to rapidly map relaxation times in studies requiring dynamic scanning, in which the presence of fat is problematic.

## 1 INTRODUCTION

Fast and accurate tissue relaxation time measurements in the presence of significant amounts of fat are particularly relevant to muscle studies, but are challenging due to the known biases in the values obtained. These measurements have been suggested to provide pathophysiological information associated with skeletal muscle injury and diseases.^1^ Normal and abnormal physiology can also be studied by monitoring the T_1_ and T_2_ recovery curves after exercise.^2^, ^3^, ^4^, ^5^, ^6^ Many of the quantitative studies use an MR sequence with variable TEs (T_2_‐prepared SSFP or fast/turbo spin echo [MSE]) for T_2_ mapping.^2^, ^3^, ^4^, ^5^, ^6^ The scan time for these conventional scan techniques is relatively long (1‐4 minutes) and limits the temporal resolution that can be achieved in assessing the recovery curves. Recently, it was demonstrated that it is possible to combine T_2_ and T_1_ measurements in a sequential order (about 2 minutes) using a modified Look‐Locker technique for T_1_ mapping.^2^ However, for experiments in which the recovery process is very fast, even shorter scan times are desired. In addition, measuring tissue parameters individually increases the risk of geometrical parameter mismatch. Therefore, there is a need for a fast acquisition scheme that encodes T_1_ and T_2_ simultaneously, addressing also the fat signal as a confounding factor in the data analysis.

Magnetic resonance fingerprinting (MRF) is a quantitative imaging technique that can map multiple relaxation times simultaneously.^7^ It often uses an efficient sampling scheme such as the spiral, which allows parameter mapping from heavily undersampled data. One of the drawbacks of the spiral sampling scheme is its sensitivity to off‐resonance effects compared with Cartesian sampling.^8^ In particular, the fat signal, with the resonance frequency of its main peak about 3 ppm offset from water, is blurred by the spiral readout. This effect is stronger when long acquisition windows are used for boosting the spiral sampling efficiency. The blurred fat signal, whose T_2_ is much longer than the T_2_ of muscle, artificially increases the “apparent” T_2_ values in the muscle regions. However, even for very short spiral acquisition trajectories, for which the chemical shift blurring effect is small, the fat signal in the muscle is known to artificially increase muscle T_2_.^9^ In a similar way, the fat signal artificially decreases the estimated muscle T_1_ value compared with its true water value.^10^ Therefore, for accurate quantification the fat signal needs to be removed, suppressed, or taken into account before accurate quantification can be achieved. One option is to remove the fat signal by applying appropriate fat suppression pulses. A drawback of this approach is that the fat suppression is sometimes incomplete,^11^ and RF pulses can result in substantial, undesired magnetization transfer effects on the water signal quantification.^12^ Moreover, in certain pathology‐related studies such as Duchenne muscular dystrophy, fat contains valuable information^13^ that can potentially be captured from the same MRF scan.

In MRF, variable TEs have been introduced as a way to encode the chemical shift of fat.^14^, ^15^, ^16^ By generating a large dictionary that is a linear combination of water and fat dictionaries, the water and fat signals can be separated in the matching process.^15^, ^17^, ^18^, ^19^ However, these dictionaries can grow very large, which may increase the risk of calculating false‐positive matches. To reduce the degrees of freedom, one could pre‐estimate the T_1_ and T_2_ of fat based on the subcutaneous fat signal, such as also done in standard quantitative approaches,^20^ but the relaxation times for subcutaneous fat may not necessarily be representative for fat in the muscle,^21^ and may therefore lead to bias in the quantitative maps. Another approach to lower the degrees of freedom in the matching process was adopted by Cencini et al, who used RF spoiling to reduce the sensitivity to T_2._ This allowed them to eliminate T_2_ as matching parameter from the dictionary,^19^ but T_2_ quantification is important in muscle studies. Ostenson et al circumvented this problem by separating water from fat before the matching process, although in a rather complicated framework.^22^

In this work we aim to improve relaxation time quantification (T_1_ and T_2_) in muscle in the presence of fat. We introduce a simple water–fat separation approach for MRF, in which the dictionary size and matching algorithm remain unchanged compared with traditional MRF. We use an alternating in‐phase/out‐of‐phase TE pattern to encode the chemical fat shift in the MRF acquisition, such that it can be combined with the well‐established 2‐point DIXON technique to separate water from fat signals.^23^ Furthermore, this approach allows water–fat separation based on a single‐run MRF scan that can help increase temporal resolution and reduce the risk of data corruption by motion or system‐related inconsistencies. The MRF image series are first reconstructed followed by conjugate phase reconstruction (CPR), using a field map measured in advance. By doing this, the 2‐point DIXON technique can be applied directly without having to introduce the *B*_0_ map as an extra degree of freedom in the system of equations to solve. The accuracy of the method is first demonstrated in computer simulations and phantom experiments. Parameter values in 9 healthy volunteers are compared with and without water–fat separation. To test whether the approach is capable of measuring small in vivo dynamic changes in relaxation times, measurements were performed during muscle recovery after plantar flexion exercise.

## 2 THEORY

**Figure 1:**
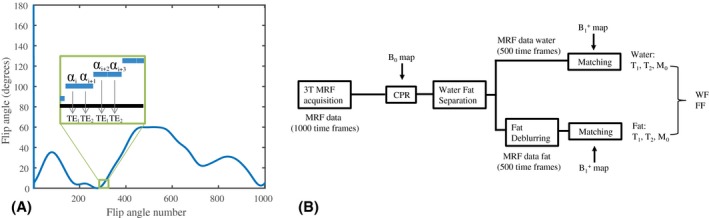
Single‐run water‐fat resolving magnetic resonance fingerprinting (MRF) sequence and image processing pipeline. A, The MRF train, similar to the one used in Sommer et al,^54^ consists of 1000 flip angles, but each 2 consecutive ones having the same value being followed by a different TE. This sequence was constructed by interleaving 2 identical flip angle trains of length 500, each having its own constant TE. The entire train is preceded by an inversion pulse seen at shot number 0. B, The 1000 MRF frames are corrected for *B*_0_ inhomogeneities by applying the conjugate phase reconstruction (CPR) using the measured *B*_0_ map and the simulated spiral k‐space trajectory as input. Subsequently, the water signal is separated from the fat signal with a 2‐point DIXON, using a 7‐peak fat model. The resulting 500 fat MRF frames are deblurred by applying CPR at a stationary frequency of 440 Hz (chemical shift offset). Water and fat (WF) MRF frames are matched individually to the same dictionary using the measured $B_{1}^{+}$ map as input, resulting in a T_1_, T_2_, and M_0_ map for water and for fat separately, which are combined into water and fat fraction (FF) maps

Before describing the experiments performed with the proposed single‐run water–fat‐resolved spiral MRF approach, which is schematically shown in Figure 1, we give a brief outline of the underlying theory regarding water–fat signal processing.

The signal intensity in a voxel **r** that contains both water, *W*(**r**), and fat, *F*(**r**), can be written as

(1) $$S\left(r,\;t\right)=\left(W\left(r\right)+F\left(r\right)\sum_{j=1}^{7}f_{j}e^{2\pi \sigma_{j}it}\right)e^{2\pi \Delta B_{0}\left(r\right)it}\;0\leq t\leq \text{TE}+\text{TA},$$

which assumes a 7‐peak fat model with normalized amplitudes *f_j_* and frequencies *σ_j_* (in hertz) as described in Ren et al.^24^ The time *t* and the readout time of the acquisition trajectory TA are in seconds, and Δ*B*_0_ is the offset in the main field (in hertz) with respect to the scanner resonance frequency. The following processing steps can be performed to obtain sharp water and fat images from 2 signals, $S_{{\text{TE}}_{1}}$ and $S_{{\text{TE}}_{2}}$, acquired with in‐phase and out‐of‐phase TEs. It should be noted that the order of the water–fat separation and the conjugate phase reconstruction steps can be reversed as well.

### 2.1 Conjugate phase reconstruction

The blurring due to the main field inhomogeneity can be corrected for by applying CPR,^25^ after which the signal equation (Equation 1) turns into

(2) $$S\left(r,\;t\right)=W\left(r\right)+F\left(r\right)\sum_{j=1}^{7}f_{j}e^{2\pi \sigma_{j}it}\;0\leq t\leq \text{TE}+\text{TA}.$$

In this process, the simulated readout trajectory can be used to generate a time map, describing at which time each k‐space position was acquired. The time map is used together with binned frequencies of the obtained *B*_0_ map to create a look‐up table of single‐frequency corrected images from which the actual corrected complex signal value is derived for each voxel.^25^

### 2.2 Water–fat separation

During the readout process (substituting *τ* = *t* − TE in Equation 2), we obtain

(3) $$S\left(r,\;\tau \right)=W\left(r\right)+F\left(r\right)\sum_{j=1}^{7}f_{j}e^{2\pi i\sigma_{j}\text{TE}}e^{2\pi i\sigma_{j}\tau }\;0\leq \tau \leq \text{TA}.$$

For the 2 TEs, Equation 3 results in the system

(4) $$\left[\begin{array}{c} S_{{\text{TE}}_{1}}\left(r,\;\tau \right)\\ S_{{\text{TE}}_{2}}\left(r,\;\tau \right) \end{array}\right]=\left[\begin{array}{cc} 1 & \sum_{j=1}^{7}f_{j}e^{2\pi i\sigma_{j}{\text{TE}}_{1}}e^{2\pi i\sigma_{j}\tau }\\ 1 & \sum_{j=1}^{7}f_{j}e^{2\pi i\sigma_{j}{\text{TE}}_{2}}e^{2\pi i\sigma_{j}\tau } \end{array}\right]\left[\begin{array}{c} W\left(r\right)\\ F\left(r\right) \end{array}\right]\;0\leq \tau \leq \text{TA}.$$

At the TE, or *τ* = 0, we obtain the system

(5) $$\left[\begin{array}{c} S_{{\text{TE}}_{1}}\left(r,\;0\right)\\ S_{{\text{TE}}_{2}}\left(r,\;0\right) \end{array}\right]=\left[\begin{array}{cc} 1 & \sum_{j=1}^{7}f_{j}e^{2\pi i\sigma_{j}{\text{TE}}_{1}}\\ 1 & \sum_{j=1}^{7}f_{j}e^{2\pi i\sigma_{j}{\text{TE}}_{2}} \end{array}\right]\left[\begin{array}{c} W\left(r\right)\\ F\left(r\right) \end{array}\right],$$

which can be solved for *W*(**r**) and *F*(**r**) using direct inversion methods.

### 2.3 Fat deblurring

During the acquisition, the accumulation of phase for 0 ≤ *τ* ≤ TA blurs the resulting fat images, following the equation $F\left(r\right)=F_{d}\left(r\right)\sum_{j=1}^{7}f_{j}e^{2\pi i\sigma_{j}\tau }$ (see Equation 4), assuming again the 7‐peak fat model with *F_d_* being the unblurred fat images. The fat images can be deblurred by correcting k‐space for the stationary off‐resonant frequencies corresponding to the different *σ_j_* in the fat model. We note that, for fat, the blurred k‐space signals *s* and the sharp k‐space signals *s_d_* are related through

(6) $$s\left(t\right)=\int \rho \left(r\right)\sum_{j=1}^{7}f_{j}e^{2{\pi i\sigma }_{j}t}e^{-ik(t)\cdot r}dr=\sum_{j=1}^{7}f_{j}e^{2\pi i\sigma_{j}t}\int \rho \left(r\right)e^{-ik\left(t\right)\;\cdot \;r}dr=\sum_{j=1}^{7}f_{j}e^{2\pi i\sigma_{j}t}s_{d}\left(t\right);$$

therefore, $s_{d}\left(t\right)=\frac{s\left(t\right)}{\sum_{j=1}^{7}f_{j}e^{2\pi i\sigma_{j}t}}.$ Hence, we can compute

(7) $$F_{d}=F^{-1}\left\{\frac{F\left\{F\right\}}{\sum_{j=1}^{7}f_{j}e^{2\pi i\sigma_{j}T}}\right\}$$

with *T* being the same time map as used in CPR, and division performed element‐wise.

For undersampled data, CPR and the fat deblurring algorithm will not correct the aliased part of the signal appropriately. However, this aliased part will later cancel out in the matching process like in spiral MRF without CPR (on‐resonance), provided that for a given spatial location the correction function applied is the same for the entire length of the MRF train,^18^, ^19^, ^26^ which is the case for CPR*.* All steps in this processing pipeline have been validated in a simulation experiment, for which the results are shown in Supporting Information Figures S1‐S3.

## 3 METHODS

### 3.1 Fingerprinting definition

A flip angle pattern of 1000 RF excitation pulses ranging from 0° to 50° was defined, preceded by an inversion pulse.^27^ A spoiled/unbalanced gradient‐echo sequence was used,^27^, ^28^ in which alternating TEs were chosen as 2.3 ms and 3.45 ms, forming 500 in‐phase and out‐of‐phase echo pairs. Within each echo pair, the flip angle was kept constant and the same spiral readout trajectory was used (see Figure 1A for this interleaved pattern). For each echo pair the starting angle of the spiral arm was rotated by 360/*N* degrees with respect to the trajectory used for the previous echo pair, with *N* being the number of spiral arms needed to fulfill Nyquist sampling. Such an acquisition results in an MRF data set with undersampling factor *N*. For smaller undersampling factors, such as *M/N*, the acquisition is repeated *M* times after a waiting time of 6 seconds for spin relaxation, this time starting with a spiral arm that is rotated by 360/*M* degrees with respect to the corresponding angle in the previous repetition of the flip angle train. The excitation RF pulse used had a time‐bandwidth product of 8, resulting in a slice profile that has been shown to have a very small effect on the parameter quantification.^29^ The RF pulse phase was kept fixed at 0°. To simplify dictionary calculations, the TR was set to a constant value of 15 ms.

### 3.2 Dictionary generation

A 3D dictionary for the 1000 RF pulses was calculated following the extended phase graph formalism,^30^, ^31^ based on the Bloch equations,^32^, ^33^ incorporating 123 645 signal evolutions.^31^ The T_1_ values ranged from 20‐100 ms in steps of 10 ms, from 100‐1000 ms in steps of 20 ms and from 1000‐2000 ms in steps of 30 ms. The T_2_ values ranged from 10‐50 ms in steps of 1 ms, from 50‐100 ms in steps of 2 ms and from 100‐500 ms in steps of 10 ms. A $B_{1}^{+}$ fraction ranging from 0.5‐1.2 in steps of 0.05 was incorporated into the dictionary calculation to account for potential local transmit gain variations resulting from wave propagation effects. Slice‐profile effects were not taken into account. Finally, for simplicity, TE variations were not taken into account. This is justified by the small difference in TEs used (1.15 ms), which would introduce a negligible change in signal amplitude due to $T_{2}^{*}$ relaxation.

### 3.3 Experimental setup

Measurements were performed on an Ingenia 3T dual transmit MR system (Philips Healthcare, Best, Netherlands).

#### 3.3.1 Phantom experiments

Phantom experiments were performed with the body coil for transmission and the 12‐element posterior and anterior coils for reception. For the phantom experiment, 5 vials containing mixtures of water and fat (0%, 20%, 30%, 40%, and 100% fat fractions) were made according to the recipe given in Hines et al^34^ and placed in a water bath with 1% salt added. For the water part, 43 mM sodium dodecyl sulphate, 43 mM sodium chloride, 3.75 mM sodium azide, and 0.3 mM Dotarem gadoteric acid were added to distilled deionized water. Two percent agar was added over heat. For the fat part, peanut oil (Jumbo, Leiden, Netherlands) was used, because its proton spectrum has been shown to be very similar to that of subcutaneous fat in human.^35^ Water and fat components were added, after which they were mixed through gentle inversion. The MRF measurements were acquired fully sampled, undersampled (R = 20), and in a noninterleaved mode. Standard T_1_, T_2_, and fat fraction (FF) mapping scans were acquired for comparison (details provided subsequently). Standard T_2_ mapping was performed with and without spectral presaturation with inversion recovery (SPIR) fat suppression to investigate the effect of fat on the T_2_ mapping analysis.

#### 3.3.2 In vivo experiments

In vivo experiments were performed with a quadrature transmit, 16‐receive element knee coil. All experiments were approved by the local medical ethics committee, and all volunteers signed an appropriate informed consent form. Two healthy volunteers were scanned to perform a comparison among fully sampled, undersampled, and noninterleaved MRF acquisitions, as well as a comparison with standard T_1_, T_2_, and FF mapping scans (details provided subsequently). The entire protocol was performed twice to obtain insight into the repeatability of the proposed technique. Nine healthy volunteers (5 males, 4 females, 24‐60 years old) were asked to perform exercise while lying in the scanner. During each exercise experiment, the subject performed concentric ankle plantar flexion (right foot) for approximately 5 minutes, while holding a rubber resistance band that was wrapped around the same foot. After this, the volunteer was asked to stay in the resting position for approximately 12 minutes. For this experiment there was no control on preworkout conditions, and the experiment was not adapted to each volunteer's maximum strength. Before the exercise experiment, a *B*_0_ map, a $B_{1}^{+}$ map, and an MRF scan were acquired. After exercise, 30 sets of interleaved MRF scans were acquired successively, each one followed by a 10‐second waiting time for spin relaxation. In 3 of the volunteers, reference T_2_ measurements were also performed, interleaved with the MRF scans. Due to the long scan time of the MSE protocol (details found subsequently), it was only possible to perform 3 reference measurements after exercise, each preceded by 4 MRF scans.

### 3.4 Magnetic resonance data acquisition

#### 3.4.1 Magnetic resonance fingerprinting scans

Interleaved MRF scans were acquired as single slice with a single spiral readout scheme using an undersampling factor of 20 and the following scan parameters: FOV = 230 × 230 mm^2^, in‐plane resolution = 1.31 × 1.31 mm^2^, slice thickness = 10 mm, spiral acquisition window = 8 ms, and scan time = 15 seconds. Additionally, noninterleaved MRF scans were acquired, in which 2 separate scans were performed directly after each other, using the same flip‐angle pattern of length 1000, but a constant TE pattern (2.3 and 3.45 ms for the first and the second train, respectively).

#### 3.4.2 *B*_0_ maps

Cartesian *B*_0_ maps were acquired matching the geometry and spatial resolution of the MRF scans, using a dual‐acquisition gradient‐echo method with a TE difference of 2.3 ms. The first TE was chosen as 2.3 ms, such that the water and the fat signals are in phase. Other scan parameters were TR = 7 ms, scan time = 2.5 seconds.

#### 3.4.3 $B_{1}^{+}$ maps

Cartesian $B_{1}^{+}$ maps were acquired for the same FOV using the dual refocusing echo acquisition mode method^36^ with the following scan parameters: in‐plane resolution = 3.28 × 3.28 mm^2^, slice thickness = 10 mm, TE_1_/TE_2_ = 1.69/2.3 ms, TR = 4.4 ms, and flip angle = α: 60°/*β*: 10° in a scan time for a single slice of less than 1 second.

#### 3.4.4 Inversion recovery turbo spin echo

Cartesian T_1_ mapping with SPIR fat suppression for comparison (in‐plane resolution = 1.31 × 1.31 mm^2^, slice thickness = 10 mm, TI = 30, 50, 100, 200, 500, 1000, 1500, and 2000 ms, TR = 5000 ms, turbo spin‐echo factor = 16, scan time = 1:05 minutes per TI).

#### 3.4.5 Multi‐turbo spin echo

Cartesian T_2_ mapping without fat suppression^37^ was used for comparison (FOV = 180 × 180 mm^2^, in‐plane resolution = 1.4 × 1.8 mm^2^, slice thickness = 10 mm, TE/ΔTE/TR = 8/8/3000 ms, 17 echoes, scan time = 3:29 minutes). In some phantom experiments this sequence was preceded by SPIR fat suppression.

#### 3.4.6 Spoiled gradient echo (DIXON)

Cartesian FF mapping was used for comparison with the following scan parameters: in‐plane resolution = 1.31 × 1.31 mm^2^, slice thickness = 10 mm, multi‐acquisition mode, TE = 4.4/5.2/5.9 ms, TR = 300 ms, flip angle = 5°, 2 averages, and scan time = 5:18 minutes. Images were reconstructed on the scanner using a 7‐peak fat model.

Because the IR and DIXON methods are currently used widely and are validated, they were chosen as reference. For T_2_, MSE was chosen because it has been validated specifically in muscle studies.

### 3.5 Processing of MRF data

All processing of the reconstructed MRF images was performed in *MATLAB* (MathWorks, Natick, MA) and run on a Windows 64‐bit machine with an Intel i7‐8700 CPU with 3.20 GHz and 64 GB internal memory. The MRF image series were processed according to the image‐processing pipeline shown schematically in Figure 1B, to obtain an MRF image series for water and for fat separately. For CPR, the frequencies in the measured *B*_0_ map were binned into steps of 3 Hz.

### 3.6 Matching of MRF data

After water–fat separation, the MRF water and fat series contained 500 time points instead of 1000. Therefore, every other time point was removed from the dictionary for 1000 RF pulses, such that the resulting dictionary describes the signal evolution for a dynamic length of 500. Here it was assumed that there is no significant signal change between successive RF pulses within an echo pair. This is similar to averaging the 2 time points within an echo pair, as done in 2‐point DIXON. The 500 separated water and fat MRF frames were matched independently to the on‐resonance dictionary, based on the largest inner product between the normalized measured signal in each voxel and the normalized dictionary entries. In this process, the measured $B_{1}^{+}$ map was used as an input to restrict the matching parameters for each voxel to T_1_ and T_2_ only. Proton density maps for the water and for the fat were calculated according to

$$M_{0}\left(x,\;y\right)=\frac{D_{m}\left(x,\;y\right)\cdot S\left(x,\;y\right)}{D_{m}\left(x,\;y\right)\cdot D_{m}\left(x,\;y\right)},$$

where $S\left(x,\;y\right)\in C^{t\times 1}$ is the non‐normalized signal and $D_{m}\left(x,\;y\right)\in C^{t\times 1}$ is the nonnormalized dictionary element corresponding to the best match in voxel‐s (*x, y*). For confirmation, water fraction (WF) and FF maps were calculated from the proton density maps following

$$\begin{array}{c} \text{WF}\left(x,\;y\right)=\frac{|M_{0}^{\text{water}}\left(x,\;y\right)|}{|M_{0}^{\text{water}}\left(x,\;y\right)\left|+|M_{0}^{\text{fat}}\left(x,\;y\right)\right|}\;\text{and}\\ \text{FF}\left(x,\;y\right)=\frac{|M_{0}^{\text{fat}}\left(x,\;y\right)|}{|M_{0}^{\text{water}}\left(x,\;y\right)\left|+|M_{0}^{\text{fat}}\left(x,\;y\right)\right|}. \end{array}$$

For validation of the MRF water–fat separation modeling, interleaved MRF results were compared with those obtained from a noninterleaved MRF experiment. With the noninterleaved approach, the basic spin history before an RF pulse at a certain time point in the MRF train is the same in the 2 data sets. The amount of acquired data is twice as large, resulting in 1000 water and 1000 fat frames after water–fat separation. Therefore, the temporal dimension of the dictionary was not reduced before matching, as opposed to the interleaved approach. For the approach without water–fat separation, only time points corresponding to a constant TE of 3.45 ms were selected from the MRF series before matching.

### 3.7 Analysis of MRF data

For each volunteer, MRF T_1_ and T_2_ maps at rest were averaged over a region of interest (ROI) in the muscle with and without water–fat separation. Two‐sided paired t‐tests were performed to assess T_1_ and T_2_ values: A *p*‐value less than 0.05 was used for statistical significance. The MRF recovery curves after exercise were produced by averaging the T_1_ and T_2_ values in an ROI of approximately 85 pixels in the muscle. The smoothness of the curves was examined in 6 volunteers. First, a smooth version of the curves was constructed by applying median filtering with a filter size of 6 time points, removing possible outliers in the data. The original MRF curves were compared with the smoothed curves by calculating the normalized residuals for each time point in the exercise curve, from which the mean and maximum values were calculated for each volunteer. Upper bounds were reported.

### 3.8 Fitting of reference T_2_ measurements

To measure the water T_2_ values of muscle in volunteers without significant fat bias, the non‐fat‐suppressed MSE data were analyzed according to the approach described in Azzabou et al^20^ using a tri‐exponential fit on the image series, discarding the first 2 TEs from a series of 17. In the first step of the fitting process, a long and a short T_2_ component of the fat and their relative amplitudes were estimated from a bi‐exponential fit in an ROI in the subcutaneous fat, which contains a negligible amount of water. In the second step, the estimated (short and long) T_2_ values and amplitudes of the subcutaneous fat were fixed in the tri‐exponential model, after which the water T_2_ value was fitted for each voxel. The reference T_2_ measurements in a phantom were also analyzed with a mono‐exponential fit (no correction for the fat signal), to facilitate comparison.

## 4 RESULTS

### 4.1 Phantom experiments

**Figure 2:**
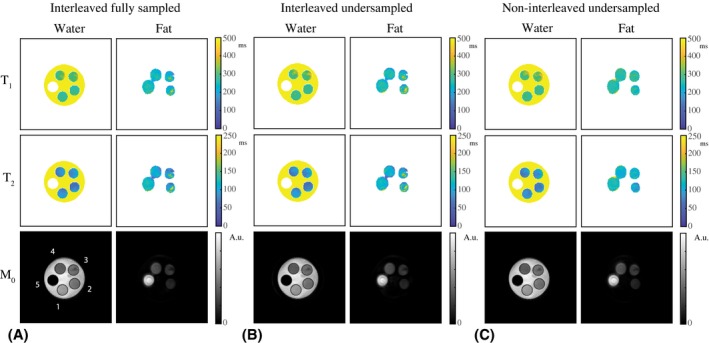
Water–fat‐resolved MRF parameter maps in a phantom. A, The T_1_, T_2_, and M_0_ maps in an interleaved fully sampled experiment are shown for the water and fat part separately. Low‐signal regions after separation were masked out in the maps. Vial numbering is shown in the M_0_ map. B, The T_1_, T_2_, and M_0_ maps from an interleaved undersampled (R = 20) experiment are of very similar quality compared with those resulting from an interleaved fully sampled experiment. C, The parameter maps in (A) and (B) are both very similar to those obtained from a noninterleaved undersampled (R = 20) experiment, in which 2 separate scans were performed, each with a constant but different TE (2.3 and 3.45 ms). The noninterleaved approach shows smaller inhomogeneities compared with the interleaved approach, which may in part be explained by the longer temporal dimension of the time‐domain signals in the first case

**Figure 3:**
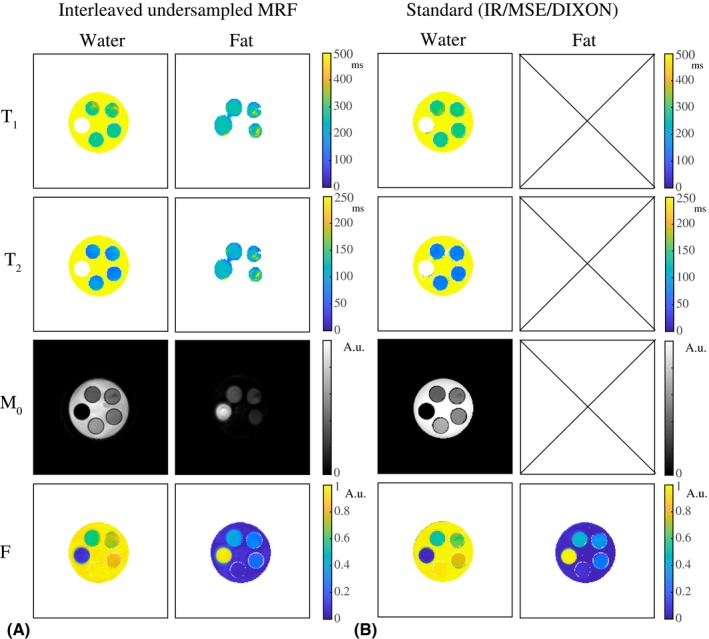
Magnetic resonance fingerprinting and reference measurements in a phantom. A, T_1_, T_2_, and M_0_ maps obtained from an interleaved, undersampled (R = 20), spiral MRF acquisition. B, Water T_1_ and M_0_ maps obtained from a fat‐suppressed inversion recovery (IR), water T_2_ maps obtained from a multiple spin‐echo (MSE) sequence with a tri‐exponential fit, and a water and fat fraction (F) map obtained from DIXON (all Cartesian). The water T_1_ maps and the water and fat fraction maps obtained with MRF are close to that obtained with fat‐suppressed IR and DIXON. The water T_2_ maps obtained with the MSE sequence show shorter values compared with those obtained from the MRF measurements. The fat T_1_, T_2_, and M_0_ maps are not shown for the standard methods, as fat suppression was used in the acquisition (IR) or during data processing (MSE)

Figure 2A shows the MRF parameter maps in an interleaved fully sampled phantom experiment. The T_1_, T_2_, and M_0_ maps are shown for the water and the fat regions separated. Figure 2B shows the same results for an interleaved undersampled experiment, and Figure 2C shows the results obtained from 2 noninterleaved separate scans with constant but different TE values for comparison. The water T_1_, water T_2_, and FF values in the 5 different vials are summarized in Table 1, indicating that the parameter maps for the interleaved fully sampled, interleaved undersampled, and noninterleaved undersampled experiments are very similar. Two‐sided paired t‐tests show no significant difference for the T_1_, T_2_, and FF values (all *p*‐values are larger than 0.05). Table 1 provides a quantitative comparison of those techniques, and includes a comparison with standard measurements, for which the parameter maps are shown in Figure 3.

**Table 1:** Comparison of water T_1_, water T_2_, and FF values for different scans in a phantom

	Water T_1_ (ms)			
	Interleaved fully sampled MRF	Interleaved undersampled MRF	Noninterleaved undersampled MRF	Fat‐suppressed IR
Vial 1	278 ± 15	289 ± 20	287 ± 21	292 ± 5.4
Vial 2	288 ± 11	288 ± 16	291 ± 22	305 ± 8.3
Vial 3	318 ± 17	325 ± 30	333 ± 28	309 ± 11
Vial 4	315 ± 22	319 ± 30	321 ± 31	316 ± 16
Vial 5	–	–	–	–

	Water T_2_ (ms)			
	Interleaved fully sampled MRF	Interleaved undersampled MRF	Noninterleaved undersampled MRF	MSE with tri‐exponential fit
Vial 1	83 ± 6.9	86 ± 8.3	80 ± 6.0	66 ± 4.1
Vial 2	77 ± 11	75 ± 11	79 ± 13	62 ± 6.9
Vial 3	86 ± 10	86 ± 14	91 ± 15	67 ± 6.2
Vial 4	79 ± 10	78 ± 12	77 ± 12	68 ± 7.7
Vial 5	–	–	–	–

	FF (%)			
	Interleaved fully sampled MRF	Interleaved undersampled MRF	Noninterleaved undersampled MRF	DIXON
Vial 1	3.3 ± 1.4	3.5 ± 1.3	2.9 ± 1.4	5.7 ± 1.1
Vial 2	22 ± 2.2	22 ± 2.4	20 ± 1.6	24 ± 1.1
Vial 3	27 ± 2.8	27 ± 2.9	28.0 ± 2.0	32 ± 3.9
Vial 4	38 ± 2.0	38 ± 1.8	39 ± 1.7	46 ± 1.3
Vial 5	96 ± 1.8	95 ± 2.0	95 ± 2.1	98 ± 0.5

Note

The phantom consists of 5 vials, each containing a different FF: 0%, 20%, 30%, 40%, and 100%. The MRF scans include an interleaved fully sampled, an interleaved undersampled (R = 20), and a noninterleaved undersampled (R = 20) experiment. Parameter values are reported as mean over an ROI in each tube ± SDs. The water T_1_, water T_2_, and FF values for an interleaved fully sampled experiment are close to that of the interleaeved undersampled experiment: 2‐sided paired t‐tests show no significant difference for the T_1_ (*P* = .1), T_2_ (*P* = 1), and FF (*P* = .5) values. This shows the robustness of the water–fat‐resolved MRF approach to undersampling. Comparison with the noninterleaved undersampled experiment shows that interleaving 2 flip angle trains introduces only minor differences: 2‐sided paired t‐tests show no significant difference for the T_1_ (*P* = .3), T_2_ (*P* = .9), and FF (*P* = .8) values. The T_1_ values obtained with standard measurements (fat‐suppressed IR for T_1_) show no significant difference compared with those obtained with MRF (*P* = 1.0), but T_2_ (*P* = 7.5·10^−3^) and FF (2.2·10^−2^) values are significantly different between MRF and standard measurements (MSE with a tri‐exponential fit for T_2_, DIXON for FF). There is a statistically significant increase in T_1_ value with increasing FF (*P* < 1^−2^), which is observed both with MRF and inversion recovery. The dependence of T_1_ on FF was previously reported in Hu and Nayak,^58^ and was attributed to a changing molecular lattice with changing FF, which leads to variations in the lattice tumbling rate and hence in T_1_ *.* Standard deviations for the fat T_1_ and T_2_ values (not reported) are slightly larger, but overall the performance is similar to that of the water component. Water T_1_ and water T_2_ values are not reported for vial 5, because this vial contained only fat. The measured FFs correspond well with the expected fat content in the different vials (0%, 20%, 30%, 40%, and 100%).

Abbrevation: IR, inversion recovery.

### 4.2 Volunteer experiments

**Figure 4:**
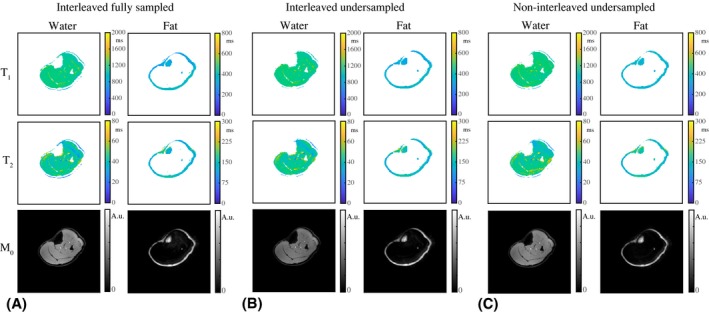
Water–fat‐resolved parameter maps in a volunteer at rest. A, The T_1_, T_2_, and M_0_ maps in a fully sampled experiment are shown for the water and the fat part separately. Low‐signal regions after separation were masked out in the maps. B, The T_1_, T_2_, and M_0_ maps from an undersampled (R = 20) experiment are of very similar quality compared with those resulting from a fully sampled experiment. C, The parameter maps in (A) and (B) are both very similar to those obtained from an undersampled (R = 20) experiment, in which 2 separate scans were performed, each with a constant TE (2.3 and 3.45 ms). Note that results in (A)‐(C) are all obtained from separate scans, in which any type of motion may have had different effects

**Figure 5:**
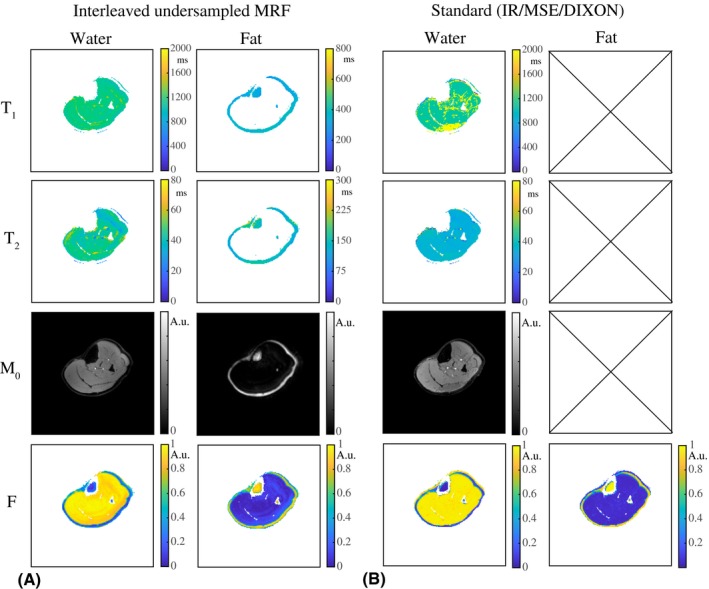
Comparison with reference measurements in a volunteer at rest. A, T_1_, T_2_, and M_0_ maps obtained from an interleaved undersampled (R = 20) spiral MRF acquisition. B, Water T_1_ and M_0_ maps obtained from a fat‐suppressed IR, water T_2_ maps obtained from an MSE sequence with a tri‐exponential fit, and water and fat fraction (F) maps obtained from DIXON (all Cartesian). The water T_1_ map obtained with MRF is close to that obtained with fat‐suppressed IR. The T_1_ map obtained with IR shows a bright region, for which the fat‐suppression pulse was probably not fully effective. The water T_2_ maps obtained with the MSE sequence show shorter values compared with those obtained from the MRF measurements. Note that the fat‐suppressed reference measurements do not deliver information about the fat

Figure 4 shows a comparison of the parameter maps in an interleaved fully sampled experiment, an interleaved undersampled experiment and a noninterleaved undersampled experiment in 1 volunteer. The parameter values averaged over ROIs in the gastrocnemius medialis (GM) muscle (Figure 7), the subcutaneous fat, and the bone marrow provide a quantitative comparison (Table 2). The MRF water T_1_/T_2_ values of muscle show differences of less than 1.8/4.3%. Table 2 also includes a comparison with standard measurements, for which the parameter maps are shown in Figure 5. Supporting Information Table S2 indicates the high repeatability in 2 volunteers: T_1_/T_2_/FF values show a maximal difference with respect to the first scan of 4.3/6.5/3.8% for MRF and 2.2/5.6/3.3% for standard measurements.

**Table 2:** Comparison of T_1_, T_2_, and FF values for different scans in vivo in 1 volunteer

	T_1_ (ms)			
	Interleaved fully sampled MRF	Interleaved undersampled MRF	Noninterleaved undersampled MRF	Fat‐suppressed IR
Muscle	1191 ± 58	1201 ± 55	1212 ± 54	1112 ± 17
Subcutaneous fat	362 ± 6.3	359 ± 8.1	349 ± 10	–
Bone marrow	332 ± 16	320 ± 7.9	359 ± 7.2	–

	T_2_ (ms)			
	Interleaved fully sampled MRF	Interleaved undersampled MRF	Noninterleaved undersampled MRF	MSE with tri‐exponential fit
Muscle	46 ± 4.5	48 ± 4.3	48 ± 4.1	35 ± 0.7
Subcutaneous fat	158 ± 7.1	164 ± 9.6	167 ± 8.7	–
Bone marrow	133 ± 15	151 ± 8.4	165 ± 7.9	–

	FF (%)			
	Interleaved fully sampled MRF	Interleaved undersampled MRF	Noninterleaved undersampled MRF	DIXON
Muscle	5.0 ± 3.2	5.3 ± 3.4	5.9 ± 2.4	3.7 ± 0.5
Subcutaneous fat	90 ± 5.7	89 ± 5.7	89 ± 6.1	91 ± 2.8
Bone marrow	92 ± 2.8	91 ± 3.4	93 ± 4.2	98 ± 1.1

Note

The MRF scans include an interleaeved fully sampled, an interleaved undersampled (R = 20), and a noninterleaved undersampled (R = 20) experiment. Parameter values are reported as mean over an ROI in each tissue region ± SDs. The T_1_, T_2_, and FF values for an interleaved fully sampled experiment are close to that of the interleaved undersampled experiment: 2‐sided paired t‐tests show no significant difference for the T_1_ (*P* = .8), T_2_ (*P* = .2), and FF (*P* = .3) values. This shows the robustness of the water–fat‐resolved MRF approach to undersampling. Comparison with the noninterleaved undersampled experiment shows that interleaving 2 flip‐angle trains introduces only minor differences: Two‐sided paired t‐tests show no significant difference for the T_1_ (*P* = .5), T_2_ (*P* = .3), and FF (*P* = .3) values. Parameter values obtained with standard measurements (fat‐suppressed IR for T_1_, MSE with a tri‐exponential fit for T_2_, DIXON for FF) are close to that obtained with MRF, except for water T_2_ in muscle, which is measured to be longer with MRF. Two‐sided paired t‐tests show no significant difference for the FF (*P* = .4). Note that T_1_ and T_2_ values reported in the subcutaneous fat and the bone marrow are fat values, whereas T_1_ and T_2_ values reported in muscle are water values. For IR and MSE sequences, T_1_ and T_2_ values in the subcutaneous fat and the bone marrow were not reported, because fat suppression was used in the acquisition (inversion recovery) or during data processing (MSE).

**Figure 6:**
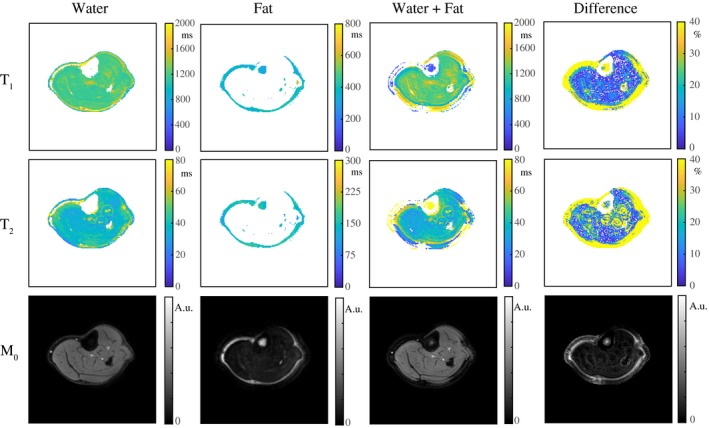
Water‐fat resolved MRF parameter maps in a volunteer's calf. The T_1_, T_2_, and M_0_ maps in an undersampled (R = 20) experiment are shown with separation (water, fat) and without separation for the out‐of‐phase TE (water + fat) in a volunteer at rest. The percentage difference between the water maps and the water + fat maps for all volunteers indicate that by separating the fat signal from the water signal, the mean estimated T_1_/T_2_ values in a region of interest in the gastrocnemius medialis muscle are significantly increased/reduced by 105 ± 94/14 ± 6 ms, underlining that fat is a confounding factor in the quantification. Low‐signal regions were masked out in the T_1_ and T_2_ maps

Figure 6 shows the parameter maps in 1 of the volunteers at rest, with and without separation of the water and the fat signal. By separating the fat signal from the water signal, the mean estimated T_2_ values in an ROI in the GM muscle are significantly reduced (*P* < .01) from 57 ± 10 ms to 43 ± 5 ms (difference of 14 ± 6 ms). The mean T_1_ values in an ROI are significantly increased (*P* < .01) from 1120 ± 68 ms to 1225 ± 64 ms (difference of 105 ± 94 ms). Standard deviations describe the variation in mean relaxation times over different volunteers. The corrected T_2_/WF (85% ± 5%) values in the muscle are slightly higher/lower compared with literature values, and the corrected T_1_ values in the muscle are in good agreement with literature.^38^, ^39^, ^40^ In the bone marrow, the water T_1_ value is measured as 327 ± 7 ms, the water T_2_ value as 155 ± 9 ms, and the FF as 85% ± 2%, agreeing with literature.^41^

**Figure 7:**
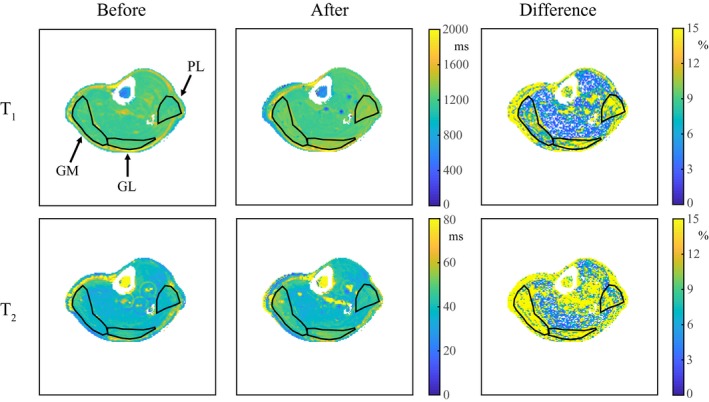
Water T_1_ and T_2_ MRF maps in a volunteer's calf before and after exercise. The water T_1_ and the T_2_ maps show an increase of approximately 65 ms and 9 ms, respectively, directly after exercise. The percentage difference between the parameter maps before and after exercise (with respect to at rest) shows that this increase is most pronounced in the gastrocnemius medialis (GM), the gastrocnemius lateralis (GL), and the peroneus longus (PL), whereas water T_1_ and T_2_ values in other muscles are mostly unchanged. Circular flow artifacts are visible around the larger vessels

Figure 7 shows the water T_1_ and T_2_ maps before and directly after exercise and their percentage difference maps (with respect to at rest) in 1 volunteer. The water T_1_ and T_2_ values increase during exercise in the GM, the gastrocnemius lateralis, and the peroneus longus, indicated by the black arrows. In the GM, the increase in water T_1_ and T_2_ is approximately 65 ms and 9 ms, respectively. Circular flow artifacts are visible around the larger vessels.

**Figure 8:**
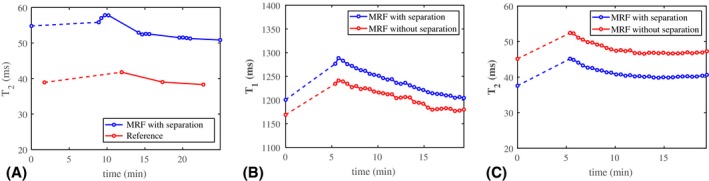
The MRF T_1_ and T_2_ measurements with and without separation and reference T_2_ measurements before and after exercise. A, The recovery curve of water T_2_ (in milliseconds) from MRF (blue) and MSE measurements (red) averaged over a region of interest in the GM in 1 volunteer. There is a systematic difference between the water T_2_ values measured with the 2 techniques, but the offset is more or less constant over time. Hence, the recovery curve measured with MRF follows the same trend as the curve measured with the reference protocol. The dashed line indicates the period during which exercise was performed. B,C, The MRF measurements with and without water–fat separation before and after exercise. The recovery curves of T_1_ (B) and T_2_ (C) (in milliseconds) averaged over a region of interest in the GM in another volunteer. With water–fat separation (blue), T_1_ increases by approximately 35 ms and T_2_ decreases by approximately 6 ms compared to without water–fat separation (red). These changes are observed along the entire curves, showing the systematic error in the presence of fat. The recovery curves show smooth behavior. The dashed line indicates the period during which exercise was performed

Figure 8A shows the recovery curves of water T_2_‐averaged over an ROI in the GM in 1 volunteer, obtained from water–fat‐resolved interleaved MRF and reference measurements. Values measured with MRF are consistently higher than those measured with the MSE approach.^20^ However, the offset is constant over time, resulting in a similar recovery trend. The constant offset varies among the 3 volunteers (7‐13 ms), as shown in Supporting Information Figure S4. This offset is also observed for the phantom experiments found in Supporting Information Table S1.

Figure 8B,C shows the MRF recovery curves of water T_1_‐averaged and T_2_‐averaged over an ROI in the GM for another volunteer at high temporal resolution with and without water–fat separation. The dashed line segment indicates the period during which exercise was performed. The water–fat separation results in a more or less constant increase in T_2_ and decrease in T_1_ over time. The curves for water T_1_ and T_2_ show smooth behavior, confirmed by very small normalized residuals after fitting (mean/maximum residual smaller than 0.2/1.1% for T_1_ and smaller than 0.8/6.5% for T_2_). For all volunteers, the water T_1_ and T_2_ values increase after exercise, and for most of the volunteers these values slowly decrease in time to the value measured before exercise (Supporting Information Figure S6).

## 5 DISCUSSION

Phantom experiments showed that the fat signal can be separated from the water signal in a single‐run MRF sequence, with an FF error in the range of 10%. Measurements in healthy volunteers showed that, using this technique, measured muscle water T_1_ values are increased and water T_2_ values are decreased compared to MRF without water–fat separation. Because fat is known to increase global T_2_ values in muscle,^9^ these results suggest that this approach improves relaxation time quantification in spiral MRF in the presence of fat, removing the bias. This was achieved without increasing the dictionary size or compromising the stability of the matching framework. In addition, this technique does not rely on assumptions about the T_1_ and T_2_ values of fat, but estimates them in the matching process. The scan time for such a single‐run MRF sequence is 15 seconds, which offers the opportunity to monitor dynamic changes in MR parameters for water and fat individually. The particular in vivo example that we used to test the performance of this approach was used to measure relaxation times during muscle recovery after exercise with high temporal resolution. The recovery curves for water T_1_ and T_2_ are smooth and display the same dynamics as recovery, using a standard and much slower sequence, showing the robustness of the approach to noise and the high stability of the postprocessing pipeline.

In this study we found that MRF systematically estimates a higher water T_2_ value in muscle compared to the reference method (MSE sequence with a tri‐exponential fit) and to literature spectroscopy and MSE values.^42^, ^43^ This difference may in part be attributed to the higher sensitivity of MRF to in‐flow and perfusion compared with the MSE sequence caused by the large number of applied excitation pulses and the long MRF train duration. Apart from that, both MRF and MSE are sensitive to outflow. However, the exact source of the T_2_‐estimation mismatch needs further investigation. In our phantom experiments, MRF also resulted in larger water T_2_ values than the reference method. In that case, however, MRF water T_2_ values are in close agreement with those obtained from a fat‐suppressed (SPIR) MSE sequence using a mono‐exponential fit (Supporting Information Table S1). These results suggest that, while the reference approach has shown good performance in vivo,^20^, ^37^ the tri‐exponential fit is the reason for underestimation of T_2_ values in our phantom data. This may be explained by the much longer water T_2_ values in this phantom than in muscle, making the tri‐exponential fitting problem harder to solve. In our current study the difference or offset between MRF T_2_ and MSE T_2_ varies between volunteers (7‐13 ms), with the largest offsets for the volunteer with the highest water T_2_ baseline value (Supporting Information Figure S4). As a consequence, MRF measurements show larger differences in baseline water T_2_ values compared with the reference measurements. One explanation could be that MRF is more sensitive to certain physiological processes such as perfusion and diffusion, emphasizing the differences among volunteers, but this hypothesis needs to be investigated further. It could also be that these differences arise from the pre‐estimation of T_2_ in the subcutaneous fat used for fitting the water T_2_ values in the reference measurements.^20^ The pre‐estimation was performed individually for each volunteer, even though the short and the long T_2_ components can become spatially dependent due to measurement imperfections such as local transmit field inhomogeneities. The measured water T_1_ and T_2_ baseline values are in the same range for all volunteers (Supporting Information Figure S5), but in some cases the water T_1_ and/or T_2_ values are clearly higher than in others. These variations are observed both with MRF and reference measurements, and may be assigned to physiological differences among the volunteers, or the noncontrolled preworkout conditions. Flow suppression may help to reduce the intensity of the circular structures that are visible in the MRF T_2_ maps around the larger vessels.

Previous exercise studies focused on simple changes in signal intensity in $T_{2}^{*}$‐weighted or T_2_‐weighted images because of the very short scan times (approximately 1 second) needed to capture dynamic changes.^44^, ^45^, ^46^ The downside of this approach is that results are not quantitative, meaning that the activation of a muscle cannot be related to specific physiological processes. Therefore, quantitative measurements are highly desirable. Previous quantitative studies have measured changes either in T_1_ or in T_2_, but not both, due to the long scan time of existing protocols, particularly in dynamic applications. In this work we were able to quantify T_1_ and T_2_ changes simultaneously at multiple timepoints before full recovery after exercise. This multiparametric dynamic approach has the potential to help disentangle different physiological processes affecting T_2_, such as changes in pH and CO_2_ levels. The dynamics measured with MRF are in agreement with previously published literature.^2^, ^47^, ^48^, ^49^ After exercise, all volunteers showed an increase in both water T_1_ and T_2_ (Supporting Information Figure S6). One of the limitations of the current study is that there was no standardization or measurement of the degree of exercise, nor control of pre‐exercise conditions. This probably explains in part why the relaxation times of some volunteers returned faster toward full recovery than others. In support of this hypothesis, some volunteers mentioned muscle soreness after exercise, while others did not, and 1 volunteer started the exercise experiment already with muscle soreness. In future applications of this technique, personalized exercise experiments will be used, in which the load of the exercise is monitored and adapted to the volunteer's maximum muscle strength, such that MRF curves can be compared among volunteers.

Although the advantage of this MRF technique was demonstrated in this paper for an exercise experiment in which a short scan time is required, there are also applications at rest that could benefit from such a water–fat‐resolved MRF sequence. Examples of such applications are abdominal/hepatic imaging or small localized regions such as the ocular muscles, in which the fat content is relatively high, which spoils the accuracy of parameter quantification in the case of spiral sampling and long readout times. Other potential applications are neuromuscular diseases such as Duchenne muscular dystrophy and other muscular dystrophies, in which fat infiltration is a hallmark of the disease, and in which T_2_ and FF are currently obtained from different scans. Further research is needed to investigate whether the method proposed provides sufficient accuracy for parameter mapping in these applications.

There are a number of ways, outlined in the next paragraphs, in which the approach presented here could be further developed. In this study, the *B*_0_ map is measured with the scanner once and used as prior information in CPR to correct for phase accumulation due to field inhomogeneities in all MRF images. As such, it enables water–fat separation based on 2 different TEs. This approach is efficient and works well if the main magnetic field, or the position of the subject, does not change over time. However, in longer experiments the main field may drift due to heating of the gradient coils, and small displacements of the volunteer may occur. An alternative approach would be to extend the number of different TEs to 3,^50^ and to estimate a *B*_0_ map for each echo triplet in the water–fat separation process using an iterative type of reconstruction,^51^ which can then be used as temporally dependent *B*_0_ input in CPR. For highly accurate (about 1% error) FF quantification, further development of the proposed technique is required, such as using a greater number of TEs,^52^ but the initial FF results in this study show the potential for accurate FF quantification with MRF in the future. The phantom experiments in this work showed larger vials in the fat channel compared with in the water channel. This can potentially be explained by a lower phantom temperature compared with body temperature, and therefore a smaller frequency shift between water and fat, decreasing the accuracy of the measured *B*_0_ map, the accuracy of the water–fat separation model, and the performance of the fat deblurring algorithm. Future improvements may include temperature measurements to address this aspect in the processing pipeline. Furthermore, interleaved MRF results in the phantom showed slightly larger inhomogeneities compared with the noninterleaved experiment. This may be explained in part by the longer temporal dimension of the time signal curves for the latter case. Optimization of the spiral starting angle pattern and using a smoother flip angle sequence may improve the interleaved MRF results. Finally, estimation of water and FFs can be improved by correcting for the noise level, especially for high water or high FF regions.^53^

The single‐run MRF scan time is determined by the number of flip angles in the MRF train. Currently, each flip angle is applied twice, each one followed by a different TE. It would be more time‐efficient if the alternating TE pattern could be incorporated only in the beginning of the MRF sequence, reducing the total number of flip angles. An estimate of the water and FFs could be derived from the (shorter) alternating TE period, which can then be used as prior information in the dictionary generation. However, such an approach requires an assumption about the T_1_ and T_2_ value of fat, whereas it has been shown that fat cannot be accurately described by a single T_1_ and T_2_ value.^24^ Therefore, we prefer to encode the water–fat shift along the entire MRF sequence. Sequence optimization, however, may offer a way to reduce the number of flip angles and hence scan time, while maintaining parameter accuracy and water–fat‐encoding capability.^54^, ^55^

The CPR and multipeak water–fat separation are computationally expensive processing steps that lead to a relatively long total processing time per MRF scan. In the current implementation, processing of 1 MRF scan (1000 frames) took 37 seconds for a maximum off‐resonance value of 100 Hz (excluding the matching). Code optimization and the use of parallel computing clusters may help to speed this up, which is especially useful for analyzing high temporal resolution MRF data, in which a large amount of MRF scans need to be analyzed. Additionally, a future processing pipeline may also include registration of the matched T_1_ and T_2_ maps before analyzing temporal parameter curves, correcting for possible motion between MRF scans.

Finally, in the matching process it was assumed that the separated fat signal in the 500 fat frames follows the on‐resonance signal model described by the extended phase graph formalism. This model assumes a single T_1_ and T_2_ value for fat, whereas in reality each of the multiple fat resonances peaks has its own T_1_/T_2_ value.^24^ The accuracy of the fat quantification can therefore potentially be improved by including multiple peaks and their scalar coupling into the dictionary simulation^9^ as well as in the water–fat separation model.^56^, ^57^ For many applications, however, such a high accuracy for the fat quantification may not be necessary.

## 6 CONCLUSIONS

This study showed the feasibility to separate water from fat signal in a single‐run MRF sequence. This technique can therefore be used to assess muscle recovery in exercise studies, but can find application in other real‐time demanding quantitative MRF measurements as well.

## Supporting information


**FIGURE S1** Validation of the processing steps in a simulation experiment. The MRF image series were created from the Shepp‐Logan phantom, by assigning different water T_1_/T_2_, fat T_1_/T_2_, and WF/FF values to the different compartments. Noise was added to the resulting time‐domain signal curves such that the resulting SNR was 28 dB. For each time frame, k‐space data were regridded onto spiral trajectories (used in phantom and in vivo experiments) using a NUFFT. From the fully sampled spiral k‐space data, spiral arms were selected according to the scanner's sampling pattern to simulate spiral undersampling artifacts. All images shown were obtained by first summing the MRF image series over the time dimension of the MRF train, after which the absolute value was taken. A, Simulated fully sampled MRF data set. (Left to right) After applying CPR, summation over the time dimension already shows a simplified result of water–fat separation because of the alternating in‐phase/out‐of‐phase TE pattern that cancels fat. The multipeak water–fat separation step correctly distributes the MRF signal over the water and the fat image channels, resulting in sharp water and blurred fat MRF images. Finally, the fat deblurring algorithm subsequently produces sharp fat MRF images. B, The results obtained from the fully sampled simulation experiment in (A) are of very similar quality compared with those obtained from an undersampled simulation experiment (R = 20), showing the robustness of the processing pipeline to undersampling. C, Reversing the order of the CPR and water–fat separation steps (with respect to [B]) in the processing pipeline results in the same sharp water and fat MRF images
**FIGURE S2** Validation of the matching process in a simulation experiment. A, T_1_, T_2_, and M_0_ maps in a fully sampled simulation experiment are shown for water and fat separately. The water and fat M_0_ maps were used to calculate water and fat fraction (F) maps. B, The parameter maps obtained from the undersampled simulation experiment are of similar quality compared with those from the fully sampled simulation experiment, except showing some minor residual undersampling artifacts. C, Reversing the order of the CPR and water–fat separation steps in the processing pipeline does not affect the matched parameter maps
**FIGURE S3** Quantitative evaluation of the parameter maps in a simulation experiment. Water T_1_/T_2_, fat T_1_/T_2_, and WF/FF values were obtained by averaging the parameter values in the regions of the different compartments of the Shepp‐Logan phantom. The values for the fully sampled simulation experiment are in perfect agreement with the true simulated values. The values for the undersampled simulation experiment coincide with the ones for the undersampled simulation experiment processed with the CPR and water–fat separation steps reversed, and are both in good agreement with the fully sampled results. Somewhat larger deviations are observed for the smallest structures of the Shepp‐Logan phantom, and are not related to the FF
**FIGURE S4** The MRF T_2_ and reference T_2_ measurements before and after exercise. A‐C, The recovery curves of water T_2_ (in milliseconds) from MRF measurements (blue) and MSE measurements (red) averaged over an ROI in the GM in 3 volunteers. There is a difference between the water T_2_ values measured with the 2 techniques, but the offset is constant within each volunteer. Hence, the recovery curves measured with MRF follow the same trend as the curves measured with the reference protocol. The dashed line indicates the period during which exercise was performed. Please note that the plot in (A) is identical to the plot in Figure 8A and is provided here for completeness
**FIGURE S5** The MRF water T_1_ and T_2_ values of muscle in 9 volunteers. The mean and SDs of T_1_ (A) and T_2_ (B) values in an ROI in the GM with and without water–fat separation are reported for each volunteer separately. Relaxation‐time values show a significant (*P *< .01) increase in T_1_ (105 ± 94 ms) and decrease in T_2_ (14 ± 6 ms) when using water–fat‐separated MRF. The SD of the T_1_ and T_2_ distributions in the ROI is much smaller for water–fat‐separated MRF compared with fat‐containing MRF. For the case without water–fat separation, the out‐of‐phase TEs were used in the matching process
**FIGURE S6** The MRF T_1_ and T_2_ measurements before and after exercise. The recovery curves of water T_1_ (A‐F) and water T_2_ (G‐L) (in milliseconds) averaged over an ROI in the GM in 6 volunteers. The volunteer in (F) shows a less smooth recovery curve compared with the other volunteers, possibly caused by motion. The volunteers in (D)‐(F) show incomplete recovery, and the volunteer in (C) shows minimal change in water T_1_, reporting muscle pain at the start of the exercise experiment. One of the data points in the volunteer in (L) is a clear outlier, possibly introduced by motion of one of the legs. The volunteer in (H) shows incomplete recovery, whereas the volunteer in (I) starts with a higher water T_2_ than after recovery. The dashed line indicates the period during which exercise was performed
**TABLE S1** Comparison of T_2_ values for different T_2_‐mapping approaches in a phantom. Note: The T_2_ values are reported as mean over an ROI in each tube ± SDs. The T_2_ values obtained from a non‐fat‐suppressed MSE sequence analyzed with a mono‐exponential fit increase with the FF. The same scan analyzed with a tri‐exponential fit results in more or less constant T_2_ values across the different vials, suggesting that the contribution of fat has been removed, but also results in underestimated T_2_ values compared with the T_2_ value in the 100% water vial obtained with a mono‐exponential fit. An MSE sequence with fat suppression also removes the fat bias in the T_2_ values, and the resulting water T_2_ values are close to that of the 100% water vial. Note that this approach would not be optimal in vivo, as complete fat suppression would be much harder to achieve.^11^ These results suggest that the tri‐exponential fitting method does not provide an accurate solution for our phantom, which has much longer water T_2_ values than muscle. The water T_2_ values obtained from an interleaved undersampled (R = 20) water–fat‐separated MRF scan are very close to the fat‐suppressed MSE sequence values. The T_2_ values were not reported for vial 5 (containing only fat), because fat suppression was used in the acquisition (spectral presaturation with inversion recovery [SPIR]) or during processing (water–fat‐separated MRF, tri‐exponential fit)
**TABLE S2** In vivo scans repeated twice in 2 volunteers at rest. Note: Water T_1_, water T_2_, and FF values are given for an interleaved undersampled MRF scan and standard quantitative measurements (fat‐suppressed inversion recovery for T_1_/MSE with a tri‐exponential fit for T_2_/DIXON for FF) for 2 scans (first and second) in 2 volunteers. Parameter values are reported as mean over an ROI in each tissue region ± SDs. Small differences in parameter values are observed between repetitions of the same scan, both for MRF and for standard measurements. Overall, the water T_1_, water T_2_, and FF values averaged over ROIs show high repeatability for MRF experiments: Two‐sided paired t‐tests show no significant change in T_1_/T_2_/FF values (*P* = .4/.7/.3 for MRF volunteer 1; *P* = .7/.9/.3 for MRF volunteer 2). Note that the standard experiments do not provide enough parameter values to perform statistical tests. The T_1_/T_2_/FF values show a maximal difference with respect to the first scan of 4.3/6.5/3.8% for MRF and 2.2/5.6/3.3% for standard measurements. The T_1_ and T_2_ values in the subcutaneous fat and the bone marrow are not reported for standard quantitative techniques, but fat suppression was performed during acquisition (IR) or data processing (MSE)
