# Supplementary material for: Water–fat separation in spiral magnetic resonance fingerprinting for high temporal resolution tissue relaxation time quantification in muscle
Source: Magn Reson Med. 2020 Jan 3;84(2):646–62. doi: 10.1002/mrm.28143 (PMC7217066; doi:10.1002/mrm.28143)
Supplement: Supplementary file 1 — FIGURE S1 Validation of the processing steps in a simulation experiment. The MRF image series were created from the Shepp‐Logan phantom, by assigning different water T1/T2, fat T1/T2, and WF/FF values to the different compartments. Noise was added to the resulting time‐domain signal curves such that the resulting SNR was 28 dB. For each time frame, k‐space data were regridded onto spiral trajectories (used in phantom and in vivo experiments) using a NUFFT. From the fully sampled spiral k‐space data, spiral arms were selected according to the scanner's sampling pattern to simulate spiral undersampling artifacts. All images shown were obtained by first summing the MRF image series over the time dimension of the MRF train, after which the absolute value was taken. A, Simulated fully sampled MRF data set. (Left to right) After applying CPR, summation over the time dimension already shows a simplified result of water–fat separation because of the alternating in‐phase/out‐of‐phase TE pattern that cancels fat. The multipeak water–fat separation step correctly distributes the MRF signal over the water and the fat image channels, resulting in sharp water and blurred fat MRF images. Finally, the fat deblurring algorithm subsequently produces sharp fat MRF images. B, The results obtained from the fully sampled simulation experiment in (A) are of very similar quality compared with those obtained from an undersampled simulation experiment (R = 20), showing the robustness of the processing pipeline to undersampling. C, Reversing the order of the CPR and water–fat separation steps (with respect to [B]) in the processing pipeline results in the same sharp water and fat MRF images FIGURE S2 Validation of the matching process in a simulation experiment. A, T1, T2, and M0 maps in a fully sampled simulation experiment are shown for water and fat separately. The water and fat M0 maps were used to calculate water and fat fraction (F) maps. B, The parameter maps obtained from the undersam [file MRM-84-646-s001.docx]

**Supporting Information Tables**

**Supporting Information Table S1.** Comparison of T_2_ values for different T_2_ mapping approaches in a phantom. T_2_ values are reported as mean over an ROI in each tube ± standard deviations. T_2_ values obtained from a non-fat suppressed MSE sequence analyzed with a mono exponential fit increase with the fat fraction. The same scan analyzed with a tri-exponential fit results in more or less constant T_2_ values across the different vials, suggesting that the contribution of fat has been removed, but also results in underestimated T_2_ values compared to the T_2_ value in the 100% water vial obtained with a mono-exponential fit. An MSE sequence with fat suppression also removes the fat bias in the T_2_ values, and resulting water T_2_ values are close to that of the 100% water vial. Note that this approach would not be optimal in vivo because complete fat suppression would be much harder to achieve (11). These results suggest that the tri-exponential fitting method does not provide an accurate solution for our phantom, which has much longer water T_2_ values than muscle. The water T_2_ values obtained from an interleaved undersampled (R=20) water-fat separated MRF scan are very close to the fat suppressed MSE sequence values. T_2_ values were not reported for vial 5 (containing only fat), because fat suppression was used in the acquisition (SPIR) or during processing (water-fat separated MRF,tri-exponential fit).

|  | **T_2_ (ms)** | | | |
| --- | --- | --- | --- | --- |
|  | **MSE with mono-exponential fit** | **MSE with tri-exponential fit** | **MSE with SPIR fat supp. and mono-exponential fit** | **Interleaved undersampled MRF** |
| **Vial 1**  **(100% water)** | 87±1.5 | 66±4.1 | 87±1.4 | 86±8.3 |
| **Vial 2** | 92±2.0 | 62±6.9 | 86±1.6 | 75±11 |
| **Vial 3** | 94±2.1 | 67±6.2 | 87±1.4 | 86±14 |
| **Vial 4** | 97±2.1 | 68±7.7 | 85±1.6 | 78±12 |
| **Vial 5 (100% fat)** | 113±1.4 | - | - | - |

**Supporting Information Table S2.** In vivo scans repeated twice in two volunteers at rest. Water T_1_, water T_2_ and fat fraction (FF) values are given for an interleaved undersampled MRF scan and standard quantitative measurements (fat suppressed IR for T_1_/MSE with a tri-exponential fit for T_2_/DIXON for FF) for two scans (1st and 2nd) in two volunteers. Parameter values are reported as mean over an ROI in each tissue region ± standard deviations. Small differences in parameter values are observed between repetitions of the same scan, both for MRF and for standard measurements. Overall the water T_1_, water T_2_, and FF values averaged over ROIs show high repeatability for MRF experiments: two-sided paired T-tests show no significant change in T_1_/T_2_/FF values (p=0.4/0.7/0.3 for MRF volunteer 1, p=0.7/0.9/0.3 for MRF volunteer 2). Note that the standard experiments do not provide enough parameter values to perform statistical tests. T_1_/T_2_/FF values show a maximal difference with respect to the first scan of 4.3/6.5/3.8% for MRF and 2.2/5.6/3.3% for standard measurements. T_1_ and T_2_ values in the subcutaneous fat and the bone marrow are not reported for standard quantitative techniques, but fat suppression was performed during acquisition (IR) or data processing (MSE).

|  | **Volunteer 1** | | | | **Volunteer 2** | | | |
| --- | --- | --- | --- | --- | --- | --- | --- | --- |
|  | **1st** | **2nd** | **1st** | **2nd** | **1st** | **2nd** | **1st** | **2nd** |
|  | **T_1_ (ms)** | | | | | | | |
|  | **MRF** | | **Fat supp. IR** | | **MRF** | | **Fat supp. IR** | |
| **Muscle** | 1201±55 | 1149±54 | 1112±17 | 1087±15 | 1171±54 | 1147±51 | 1105±15 | 1091±14 |
| **Subc. fat** | 359±8.1 | 363±7.5 | - | - | 351±10 | 360±0 | - | - |
| **Bone marrow** | 320±7.9 | 317±13 | - | - | 319±6.9 | 321±9.5 | - | - |
|  | **T_2_ (ms)** | | | | | | | |
|  | **MRF** | | **MSE** | | **MRF** | | **MSE** | |
| **Muscle** | 48±4.3 | 45±3.8 | 35±0.7 | 34±0.6 | 46±4.9 | 43±4.8 | 34±0.5 | 33±0.5 |
| **Subc. fat** | 164±9.6 | 155±6.6 | - | - | 166±7.1 | 173±8.3 | - | - |
| **Bone marrow** | 151±8.4 | 157±22 | - | - | 160±13 | 158±10 | - | - |
|  | **FF (%)** | | | | | | | |
|  | **MRF** | | **DIXON** | | **MRF** | | **DIXON** | |
| **Muscle** | 5.3±3.4 | 5.5±3.4 | 3.7±0.5 | 3.9±0.6 | 6.9±4.3 | 7.2±4.4 | 4.2±0.7 | 4.1±0.7 |
| **Subc. fat** | 89±5.7 | 90±5.7 | 91±2.8 | 88±1.2 | 91±2.8 | 92±2.5 | 91±3.6 | 90±4.7 |
| **Bone marrow** | 91±3.4 | 91±3.0 | 98±1.1 | 97±4.0 | 90±4.3 | 90±4.1 | 98±0.7 | 98±0.9 |

**Supporting Information Figures**


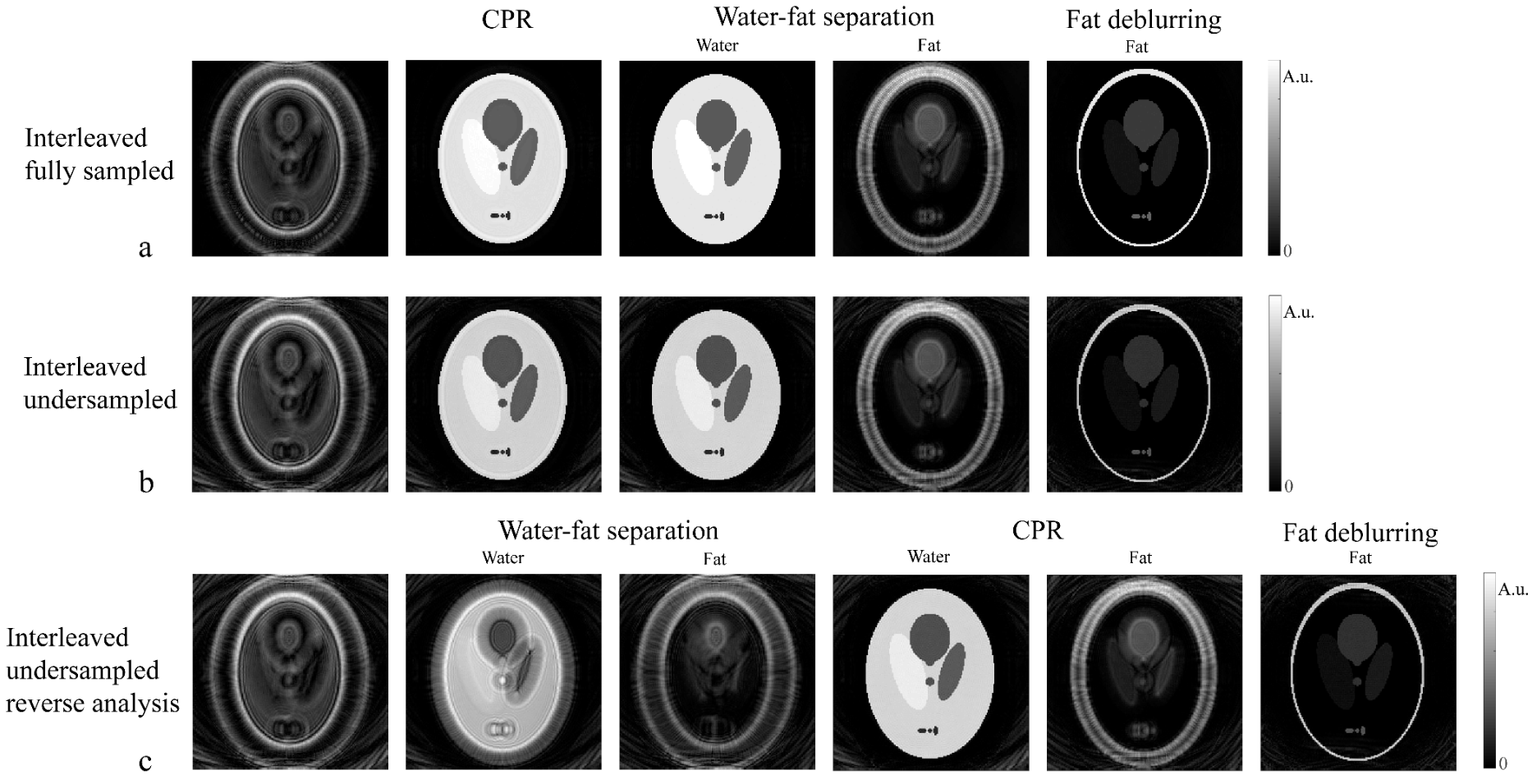


Supporting Information Figure S1. Validation of the processing steps in a simulation experiment. MRF image series were created from the Shepp-Logan phantom, by assigning different water T_1_/T_2_, fat T_1_/T_2_ and WF/FF values to the different compartments. Noise was added to the resulting time-domain signal curves such that the resulting SNR was 28 dB. For each time frame, k-space data was regridded onto spiral trajectories (used in phantom and in vivo experiments) using a NUFFT. From the fully sampled spiral k-space data spiral arms were selected according to the scanner’s sampling pattern to simulate spiral undersampling artifacts. All images shown were obtained by first summing the MRF image series over the time-dimension of the MRF train after which the absolute value was taken. (a) Simulated fully sampled MRF data set. (From left to right) After applying CPR, summation over the time dimension already shows a simplified result of water-fat separation because of the alternating in-phase/out-of-phase TE pattern that cancels fat. The multi-peak water-fat separation step correctly distributes the MRF signal over the water and the fat image channels, resulting in sharp water and blurred fat MRF images. Finally, the fat deblurring algorithm subsequently produces sharp fat MRF images. (b) The results obtained from the fully sampled simulation experiment in (a) are of very similar quality compared to those obtained from an undersampled simulation experiment (R=20), showing the robustness of the processing pipeline to undersampling. (c) Reversing the order of the CPR and water-fat separation steps (with respect to (b)) in the processing pipeline results in the same sharp water and fat MRF images.


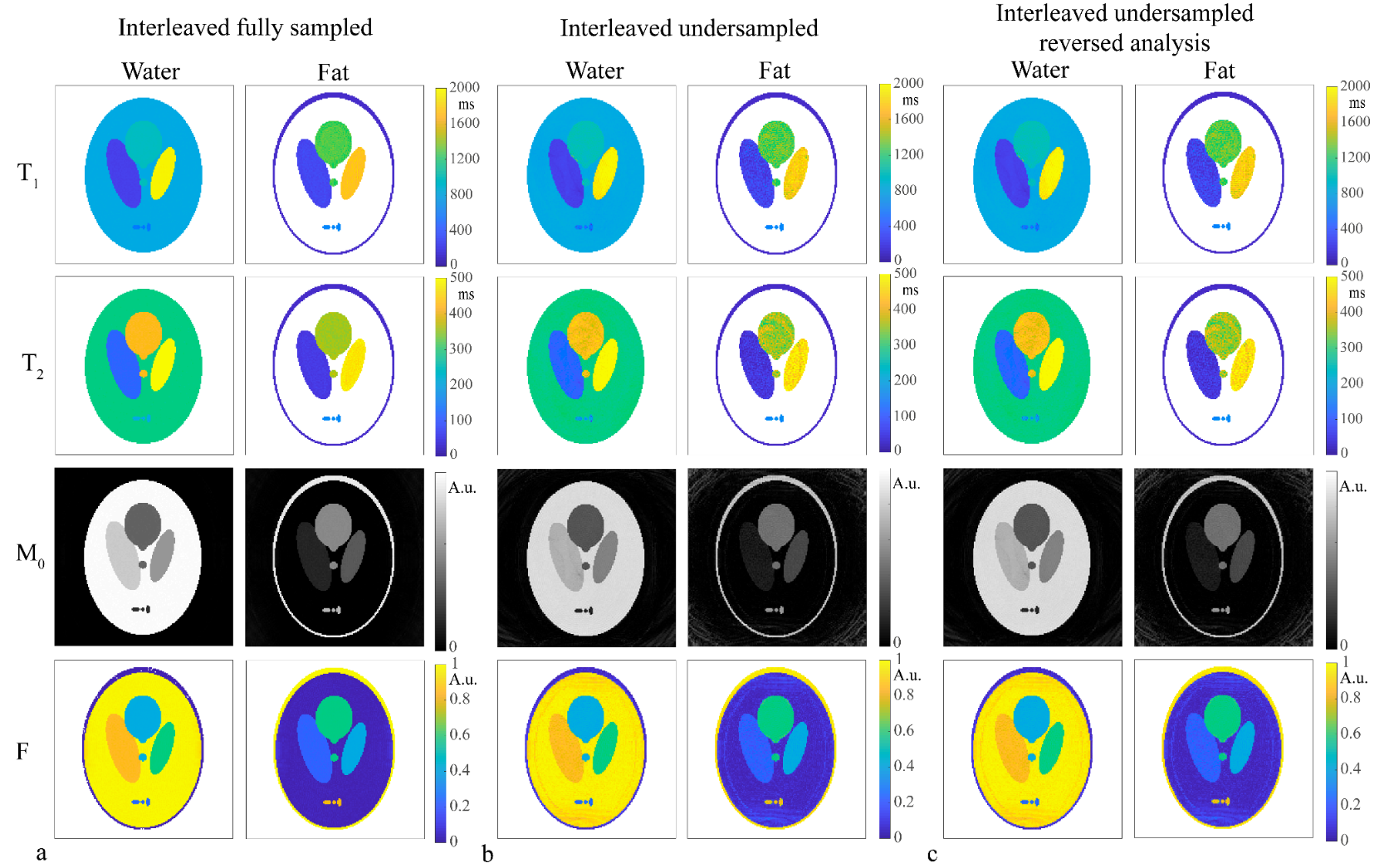


**Supporting Information Figure S2.** Validation of the matching process in a simulation experiment. (a) T_1_, T_2_ and M_0_ maps in a fully sampled simulation experiment are shown for water and fat separately. The water and fat M_0_ maps were used to calculate water and fat fraction (F) maps. (b) The parameter maps obtained from the undersampled simulation experiment are of similar quality compared to those from the fully sampled simulation experiment, except showing some minor residual undersampling artifacts. (c) Reversing the order of the CPR and water-fat separation steps in the processing pipeline does not affect the matched parameter maps.


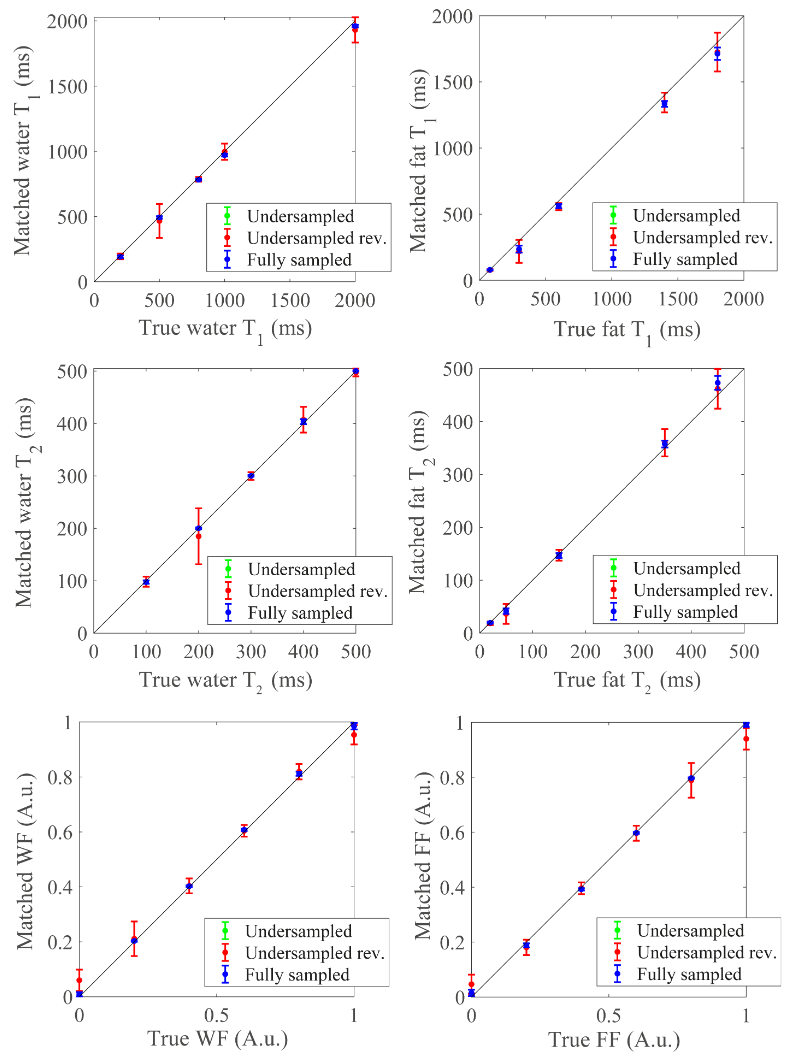


**Supporting Information Figure S3.** Quantitative evaluation of the parameters maps in a simulation experiment. Water T_1_/T_2_, fat T_1_/T_2_ and WF/FF values were obtained by averaging the parameter values in the regions of the different compartments of the Shepp-Logan phantom. The values for the fully sampled simulation experiment are in perfect agreement with the true simulated values. The values for the undersampled simulation experiment coincide with the ones for the undersampled simulation experiment processed with the CPR and water-fat separation steps reversed, and are both in good agreement with the fully sampled results. Somewhat larger deviations are observed for the smallest structures of the Shepp-Logan phantom, and are not related to the fat fraction.


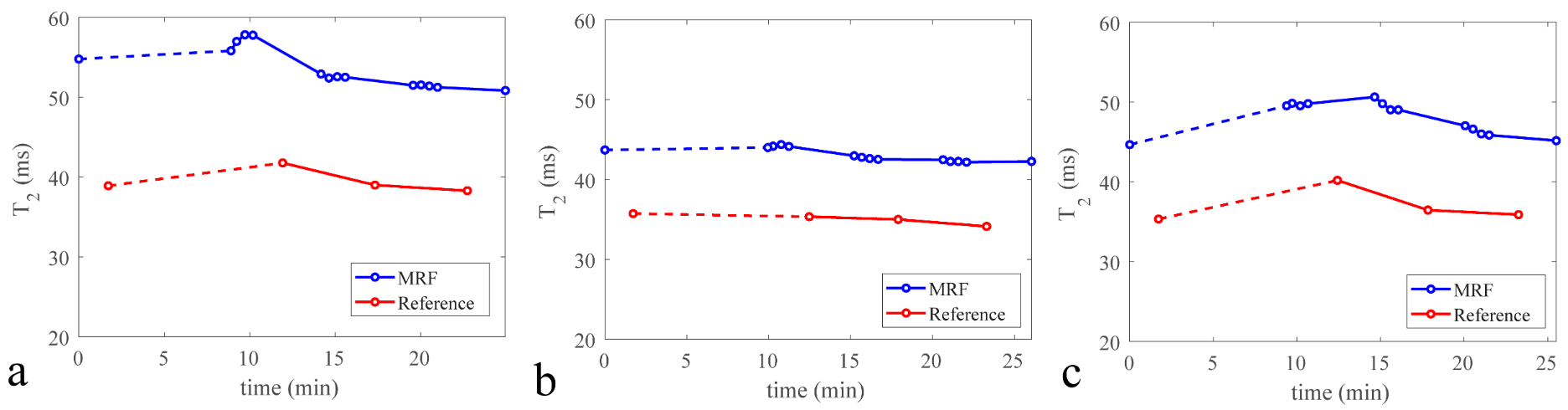


Supporting Information Figure S4. MRF T_2_ and reference T_2_ measurements before and after exercise. The recovery curves of water T_2_ in ms from MRF measurements (blue) and MSE measurements (red) averaged over an ROI in the GM in three volunteers (a, b, c). There is a difference between the water T_2_ values measured with the two techniques, but the offset is constant within each volunteer. Hence, the recovery curves measured with MRF follow the same trend as the curves measured with the reference protocol. The dashed line indicates the period during which exercise was performed. Please note that the plot in (a) is identical to the plot in Figure 8a and is given here for completeness.


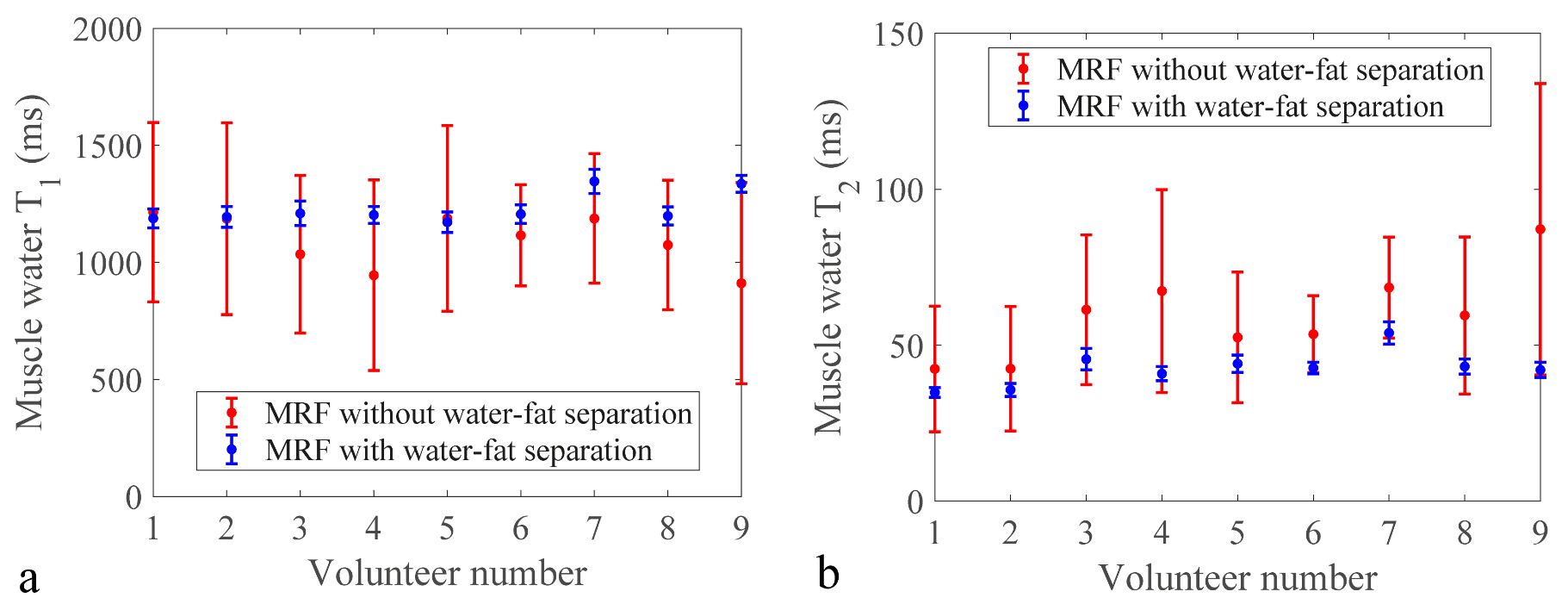


**Supporting Information Figure S5**. MRF water T_1_ and T_2_ values of muscle in nine volunteers. Mean and standard deviations of T_1_ (a) and T_2_ (b) values in an ROI in the GM with and without water-fat separation reported for each volunteer separately. Relaxation time values show a significant (p<0.01) increase in T_1_ (105±94 ms) and decrease in T_2_ (14±6 ms) when using water-fat separated MRF. The standard deviation of the T_1_ and T_2_ distributions in the ROI is much smaller for water-fat separated MRF compared to fat-containing MRF. For the case without water-fat separation the out-of-phase echo times were used in the matching process.


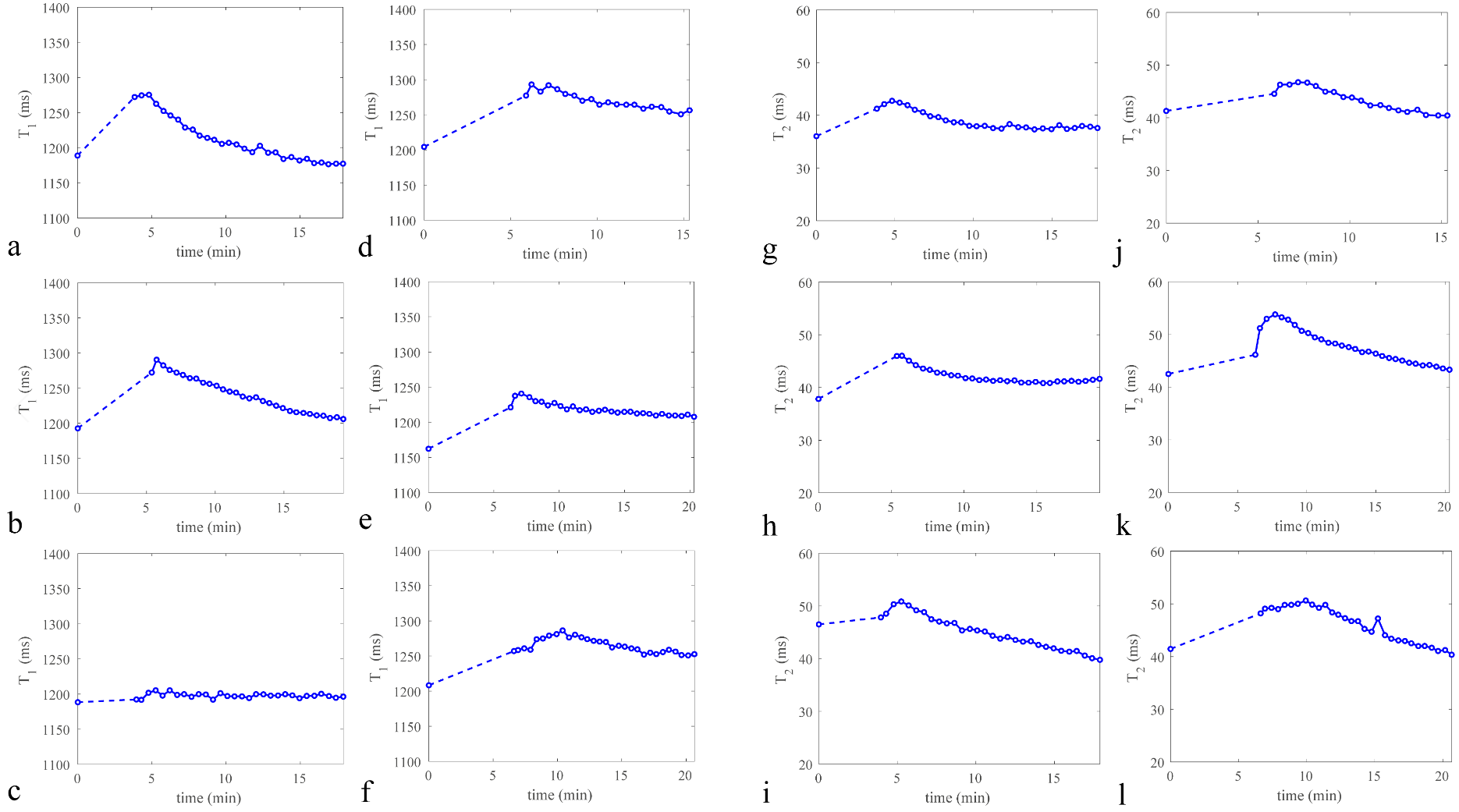


Supporting Information Figure S6. MRF T_1_ and T_2_ measurements before and after exercise. The recovery curves of water T_1_ (a-f) and water T_2_ (g-l) in ms averaged over an ROI in the GM in six volunteers. The volunteer in (f) shows a less smooth recovery curve compared to the other volunteers, possibly caused by motion. The volunteers in (d), (e) and (f) show incomplete recovery, and the volunteer in (c) shows minimal change in water T_1_, reporting muscle pain at the start of the exercise experiment. One of the data points in the volunteer in (l) is a clear outlier, possibly introduced by motion of one of the legs. The volunteer in (h) shows incomplete recovery, while the volunteer in (i) starts with a higher water T_2_ than after recovery. The dashed line indicates the period during which exercise was performed.
